# Aims & scope

**DOI:** 10.1016/j.euprot.2019.07.005

**Published:** 2019-08-13

**Authors:** Maarten Dhaenens

**Affiliations:** ProGenTomics, Laboratory of Pharmaceutical Biotechnology, Ghent University, Ottergemsesteenweg 460, 9000, Gent, Belgium

## 1 Experimental design and data analysis

Advancing knowledge requires taking risks. However, novel biology is often a prerequisite for publication, and this requirement is currently holding scientists back from taking risks. This is stalling scientific progress. Yet, every experiment that is built on a **solid experimental design** and **explored with robust data analysis** techniques is equally valuable to the advancement of knowledge. This is why EuPA Open Proteomics provides an open platform to share good experiments within the field of proteomics. Here, novel biology is a by-product of good experimentation.

EuPA Open Proteomics welcomes:

• *benchmark datasets;*
• *protocols;*
• *reviews and opinions;*
• *original research articles;*
• *heralded datasets.*

The focus is on detailed method description (wet lab) and on extensive annotation of metadata (dry lab). This in turn propagates corroboration, repeatability and alternative interpretations of methodology as well as biology. EuPA Open Proteomics endorses the *EuBIC Guidelines for Reproducible MS-based Experiments* (https://eubic.github.io/ReproducibleMSGuidelines/).

## 2 Early career researchers

EuPA Open Proteomics is making an extra effort to support early career researchers in their scientific development. The different article formats are an impulse to take risks and critically assess scientific methodology, promoting good practice and keeping an open mind on solid experimentation. Additionally, at least one junior scientist (3rd year PhD student to 5 years post PhD) is appointed as a reviewer for each submission, so that young researchers can take part in the revision process. Notably, reading a published paper to extract the relevant conclusions for your own research is very different from reading a paper to verify the robustness of the claims that were made. It opens up your mind to other perspectives and teaches you how to write a good manuscript.

If you are interested in getting involved, please fill out the form below / send an e-mail to the Managing Editor describing your experience, your research subject and providing your current position, affiliation and contact details.

## 3 Formatting requirements

There are no strict formatting requirements but all manuscripts must contain the essential elements needed to convey your manuscript, including **“Abstract”**, **“Keywords”**, **“Introduction”**, **“Materials and Methods”**, **“Results and Discussion”**, **“Conclusions”**, **Artwork** and **Tables** with Captions. EuPA Open Proteomics strongly encourages the authors to additionally include an **“Unexplored”** section before the conclusions, wherein they highlight what should be taken into consideration to correctly interpret their results. This includes unexplored validation strategies, potential future additions to the experimental design, alternative data analysis approaches, etc. This shows to both the reviewers and the readership that the authors are aware about the strengths and the weaknesses of their research. Hiding weaknesses in hopes of passing the reviewers is not in anyone’s interest.

### 3.1 Benchmark datasets

Sample preparation, data acquisition and data processing are all prone to error. Intelligent experimental design allows the thorough inspection of each step in a novel approach. Benchmark datasets are highly curated and validated datasets that contain the fundamental information to allow the verification of whether a new experimental approach gives the desired outcome. Benchmark datasets allow the elucidation of the strengths and weaknesses of new approaches, and help in figuring out where varying biological conclusions come from. For the bioinformatics community, they are a cornerstone for developing new algorithms and in time EuPA Open Proteomics aims to provide a substantial database of such datasets freely available to the bioinformatics community. Benchmark datasets/samples would also include inter-laboratory comparisons to help to understand variability.

### 3.2 Protocols

Repeatability or test-retest reliability is essential to demonstrate that the current state of knowledge is correct. This format of paper allows the presentation of new, properly validated protocols. Submissions should contain data from experiments that illustrate the benefit and applicability of the protocol, as well as a discussion of its limitations. Protocols cover every step in a proteomics workflow: sample preparation, data acquisition and computational analysis.

### 3.3 Reviews and opinions

Reviews should describe the state of the art illustrated by the recent literature, including an analysis of the current strengths, weaknesses and direction of development of the field. Alternatively, they provide an analysis of the diversity in a field. EuPA Open Proteomics especially welcomes reviews that focus on the diversity in biological conclusions. Assuming that biology is relatively coherent, contradicting biological results, at least in part, can be attributed to different experimental designs. The generation of the biological samples, the use of different analytical techniques (MS, WB, …) or the application of different data analytical strategies, all give different perspectives which not uncommonly lead to different biological conclusions. Such contributions would focus on these aspects of experimentation in an effort to figure out why a single biological phenomenon can look so diverse from different perspectives.

Opinions focus more on certain protocols or experimental designs that are not working “in your hands”. This could be due to many different reasons. The protocols should be described in a way that they pinpoint protocol misuse or overuse in the community. Opinions can contain novel data of the detailed experimental study of e.g. each step in the protocol.

### 3.4 Original research articles

The journal will accept all types of high quality primary research. This includes:

• Datasets from well-designed experiments that do not yield the hypothesized biology or methodological outcome. By making these results available publically, future researchers can look for hints of other biology or figure out why the conclusions do not match with the initial hypothesis.
• Corroboration is as important as the initial discovery. EuPA Open Proteomics welcomes manuscripts describing experiments that corroborate recently published biology. Scoops are only relevant from a commercial point of view, not from a scientific one.
• (Orthogonal) Re-use of public data can increase annotation rate and alternative quantification strategies can surface more subtle changes. This is why these efforts are welcome.
• Proteomes that are mined to increasing depths. Knowing that certain proteins are expressed in certain cell lines / tissues / organisms / conditions is essential to build a protein atlas of the biotic world. EuPA Open Proteomics welcomes contributions that dig deeper than previously described.
• Proteomics on non-model organisms is specifically hard and requires separate workflows, like proteogenomics. EuPA Open Proteomics provides a platform to share this data.

### 3.5 Heralded datasets

To illustrate the willingness of EuPA Open Proteomics to publish good experiments irrespective of their outcome, authors can e-mail their experimental design to the journal before they start the experiment. For these heralded datasets, an editor is appointed to assess the validity of the research protocol and to be the senior reviewer in the reviewing process of the article, once the data has been generated. This way, researchers can initiate their research with the promise of consideration for publication, irrespective of the biological outcome. EuPA Open Proteomics can delay the publication of the detailed description of the experimental design if authors choose to publish the biology elsewhere (to avoid issues of prior publication).

